# Morphological changes of macular neovascularization during long-term anti-VEGF-therapy in neovascular age-related macular degeneration

**DOI:** 10.1371/journal.pone.0288861

**Published:** 2023-12-22

**Authors:** Daniel Pauleikhoff, Marie-Luise Gunnemann, Martin Ziegler, Britta Heimes-Bussmann, Eike Bormann, Isabel Bachmeier, Siqing Yu, Beatriz Garcia Armendariz, Laurenz Pauleikhoff

**Affiliations:** 1 Dep. of Ophthalmology, St. Franziskus Hospital Muenster, Münster, Germany; 2 Institute of Biostatistics and Clinical Research, University Muenster, Münster, Germany; 3 F. Hoffmann-La Roche Ltd., Pharma Research and Early Development, Basel, Switzerland; 4 Dep. of Ophthalmology, Faculty of Medicine, University of Hamburg, Hamburg, Germany; Kobe University Graduate School of Medicine School of Medicine: Kobe Daigaku Daigakuin Igakukei Kenkyuka Igakubu, JAPAN

## Abstract

**Purpose:**

To analyze the morphological changes of macular neovascularization (MNV) in exudative neovascular age-related macular degeneration under long-term intravitreal anti-vascular endothelial growth factor (VEGF) therapy in a retrospective cohort study.

**Methods and patients:**

We evaluated 143 nAMD eyes of 94 patients (31 male, 63 female; initial age 55–97 y, mean age 75.9 ± 7.5 y), who started anti-VEGF therapy (IVAN pro re nata (PRN) protocol) between 2009–2018 and received ongoing therapy until the last recorded visit (mean follow-up 5.3 ± 2.9 y, range 1–14 y). The mean total number of injections was 33.3 ± 19.8 with 7.0 ± 2.3 injections/year. MNV size and, if present, associated complete retinal pigment epithelium (RPE) and outer retina atrophy (cRORA) size were measured on optical coherence tomography (OCT) volume scans at the initial visit and for each year of follow-up. MNV and cRORA were identified on B-scans and their respective borders were manually transposed onto the en-face near infrared image and measured in mm^2^.

**Results:**

MNV enlarged through follow-up, with a mean growth rate of 1.24 mm^2^ / year. The mean growth in MNV size was independent of initial MNV size, age, gender, MNV subtypes or number of injections per year. Nevertheless, a great interindividual variation in size and growth was observed. cRORA developed in association with increasing MNV size and its incidence increased linearly over follow-up. cRORA lesions also showed continuous growth by a rate of 1.22 mm^2^ / year.

**Conclusions:**

Despite frequent long-term anti-VEGF therapy, we observed ongoing MNV growth. This is consistent with the concept that the development of MNV may be a physiological biological repair mechanism to preserve RPE and photoreceptor function, provided hyperpermeability and fluid exudation are controlled. Whether recurring low VEGF levels or other factors are responsible for MNV growth remains controversial.

## Introduction

Intravitreal anti-vascular endothelial growth factor (VEGF) therapy is the most effective treatment in exudative neovascular age-related macular degeneration (nAMD) at present [1]. After the initial loading doses, an improvement in best-corrected visual acuity (BCVA) in eyes with macular neovascularization (MNV) associated with a decrease in mean central foveal thickness (CFT) on optical coherence tomography (OCT) is often observed. In the long-term, a stabilization of both function and fluid distribution can be achieved with consequent application of different treatment strategies such as IVAN scheme, CATT scheme, or Treat-and-Extent (T&E), as shown by previous reports [2–6].

However, these long-term studies focused on the recurrence of MNV hyperpermeability, lack of retinal pigment epithelium (RPE) barrier function, and fluid distribution on OCT, since these were used for retreatment decisions. The basic morphological vascular patterns of MNV were often only studied before treatment initiation, when fundus fluorescein angiography (FFA) in addition to OCT was performed and MNV subtypes were classified based on the “Consensus on Neovascular AMD Nomenclature” (CONAN) group report [7]. During follow-up and for re-treatment decisions, changes in the vascular characteristics were of minor interest. In addition, because of the difficulty to study the whole fibrovascular complex on single OCT scans, specific morphological features like subretinal hyperreflective material (SHRM), hyperreflective foci and atrophic areas and their impact on treatment outcome were investigated [2, 4, 8].

Only after the introduction of OCT-angiography (OCT-A) did the underlying vascular changes receive renewed attention. Firstly, type 1 MNV without associated exudation and negative visual effects were uncovered and termed “quiescent” MNV (also referred to as “nonexudative nAMD”) [9–11]. Secondly, Spaide et al. described differential patterns of MNV growth under anti-VEGF therapy with a maturation of central vessels and recurrent capillary growth around margins ("Bonsai" effect) [12]. Numerous subsequent studies have further specified this recurrent and differential perfusion of MNV under the different treatment strategies [13, 14].

Nevertheless, the question remains to what extent anti-VEGF therapy has an antiangiogenic effect in addition to the major anti-permeability impact and if the presence of MNV itself is damaging photoreceptors and the RPE. This was investigated in the present study by volume OCT scan analysis of long-term changes of MNV under anti-VEGF therapy. In addition, associated MNV characteristics like the presence and size of complete RPE and outer retina atrophy (cRORA; according to the Consensus Definition for Atrophy Associated with Age-Related Macular Degeneration (CAM)) [15, 16] as well as their correlation with MNV size were assessed.

## Methods

The present analysis is a single-center, retrospective cohort study of spectral domain (SD)-OCT volume scans of nAMD patients who started anti-VEGF therapy at the Department of Ophthalmology, St. Franziskus Hospital Muenster, Germany, between 2009–2018. In order to identify these patients patients with new or ongoing anti-VEGF treatment for nAMD in 2018–2019 were screened for study inclusion. The inclusion criterium was exudative nAMD with anti-VEGF-treatment and the availability of continuous follow up until the last visit with consequent implementation of OCT based retreatment criteria (IVAN PRN) at all visits. Therefore, many patients with interruptions or discontinuations because of medical conditions like major hemorrhages or because of change of treatment center were excluded. Patient data were anonymized and included until 2022 and the morphologic analysis of MNV was performed only after an uninterrupted clinical course of treatment has been established during this follow-up to limit selection bias.

All patients gave their written informed consent to anti-VEGF therapy. The need for informed consent for participation in this study was waived due to its retrospective design and anonymized data evaluation, which was approved by the local Institutional Ethics committee and adhered to the tenets of the Declaration of Helsinki for research involving human subjects. The anonymized data were accessed for research purposes starting in 2021.

The diagnosis of nAMD was confirmed by a retina specialist (D. P.) based on multimodal imaging acquired using Heidelberg Retina Angiograph 2 (HRA2) and Heidelberg Spectralis SD-OCT (both Heidelberg Engineering, Heidelberg, Germany) including obligatory FFA. The settings of the HE Spectralis SD-OCT B-scans were 6 ×6 mm OCT volume scans, consisting of 49 B-scans (appr. 125 μm between each scan) with 9 times averaging. Patients then were treated according to an “as needed” or “pro re nata” (PRN) protocol as described in the IVAN study [17]. Treatment was performed with either bevacizumab, ranibizumab or aflibercept at the treating physician’s discretion. In case of insufficient response, a switch between agents was possible.

For this study, MNV and, if present, cRORA were identified on OCT volume scans by one experienced grader (D. P.). MNV was identified on OCT B-scans based on a correlation of FFA features of MNV and OCT features such as a flat and irregular pigment epithelial detachment in case of type 1 MNV or subretinal hyperreflective material in case of type 2 MNV. For type 3 MNV only RAP with stage 3 (confirmed on FA) were included where the sub-RPE MNV lesion could also be identified on OCT volume scans. In order to measure MNV size, its borders were manually transposed onto the corresponding en-face near infrared image (NIR) and quantified using the in-built region finder software (Heidelberg Engineering, Heidelberg, Germany), a method also used in other, recently published study [17]. In both studies, MNV was measured therefore based on OCT rather than ICG angiography or OCT angiography (both were for longer follow up not available), and this approach may influence measurements. MNV size was measured at treatment initiation and on one OCT volume scan per year of follow-up.

Similarly, where present, borders of cRORA were identified on OCT B-scans by a visible band of hypertransmission into the choroid associated with subsidence of the inner nuclear layer and/or outward deviation of the outer plexiform layer, thinning or absence of the outer nuclear layer, thinning or absence of the photoreceptor inner/outer segments, or RPE–Bruch’s membrane as defined by the CAM consortium [15, 16]. The areas of cRORA developed on top of the MNV (at an area of regressed MNV–Fig 2B–or on top of an MNV–Fig 2C) were transposed onto the corresponding en-face NIR images. These cRORA areas were measured, if present, at treatment initiation and at every year of follow-up in mm^2^ by the region finder software (Heidelberg Engineering, Heidelberg, Germany).

Annual measurements of MNV and cRORA size were manually exported into Microsoft Excel (Microsoft Corporation, Redmond, WA, USA) for each patient. In cases of multiple areas of cRORA, individual areas were added up and reported as total area of cRORA.

To analyze growth over time for both MNV and cRORA, a mixed model was used with time as the categorical variable and the difference between initial size and size at each annual visit until the end of the follow-up as the dependent variable. To assess the influence of age, sex, MNV subtypes as defined by the CONAN group [7] or number of injections per year on MNV growth, linear mixed model regressions were performed. Each regression model included time-to-end of the follow-up, the initial size and one variable of interest, as well as a random intercept for the individual. To assess the relationship between MNV and cRORA size, a linear mixed model with a random intercept for the individual eye and MNV squared as the independent variable was used. A cumulative incidence function was used to visualize the risk of cRORA occurring over time. Categorical variables are presented as absolute and relative frequencies. Continuous variables are presented as mean and standard deviation (SD) as well as median and range.

Statistical analysis was performed using SAS (version 9.4, SAS Institute, Cary, North Carolina, USA) and R (version 4.3.0, https://www.r-project.org). P-values below 0.05 were regarded as statistically significant.

## Results

### Baseline characteristics

One hundred and forty-three eyes of 94 patients with nAMD (31 male, 63 female) were included in this study. Mean age at the start of treatment was 75.9 ± 7.5 years and mean follow-up was 5.3 ± 2.9 years (range 1–14 y). Mean total number of anti-VEGF injections was 33.3 ± 19.8 with a mean of 7.0 ± 2.3 injections/year. MNV subtypes were graded as type 1 in 59 eyes (41%), type 2 in 54 eyes (38%), and type 3 in 30 eyes (21%). Full baseline characteristics are reported In Table 1.

**Table 1:** Description of cohort. 143 eyes of 94 patients with exudative nAMD were included in this study. Continuous variables are presented as mean ± standard deviation. Follow-up time and initial MNV size are also characterized using minimum and maximum values as well as median. Categorical variables are shown as numerical and percentage values.

Characteristic	Data
Number of eyes	143
Number of patients	94
Baseline age (yrs), mean ± SD	75.9 ± 7.5
Sex	
Male, no. (%)	31 (33%)
Female, no. (%)	63 (67%)
Number of injections	
Overall, mean ± SD	33.3 ± 19.8
per year, mean ± SD	7.0 ± 2.3
Follow-up time (yrs), mean ± SD (range, median)	5.3 ± 2.9 (1–14, 4.0)
MNV subtypes	
Type 1, no. (%)	59 (41%)
Type 2, no. (%)	54 (38%)
Type 3, no. (%)	30 (21%)
Initial MNV size (mm^2^), mean ± SD (range, median)	6.7±5.6 (0.27–30.0, 5.2)

SD = standard deviation; MNV = macular neovascularization; nAMD = neovascular age-related macular degeneration; OCT = Optical coherence tomography.

### Changes in MNV size

**Fig 1:**
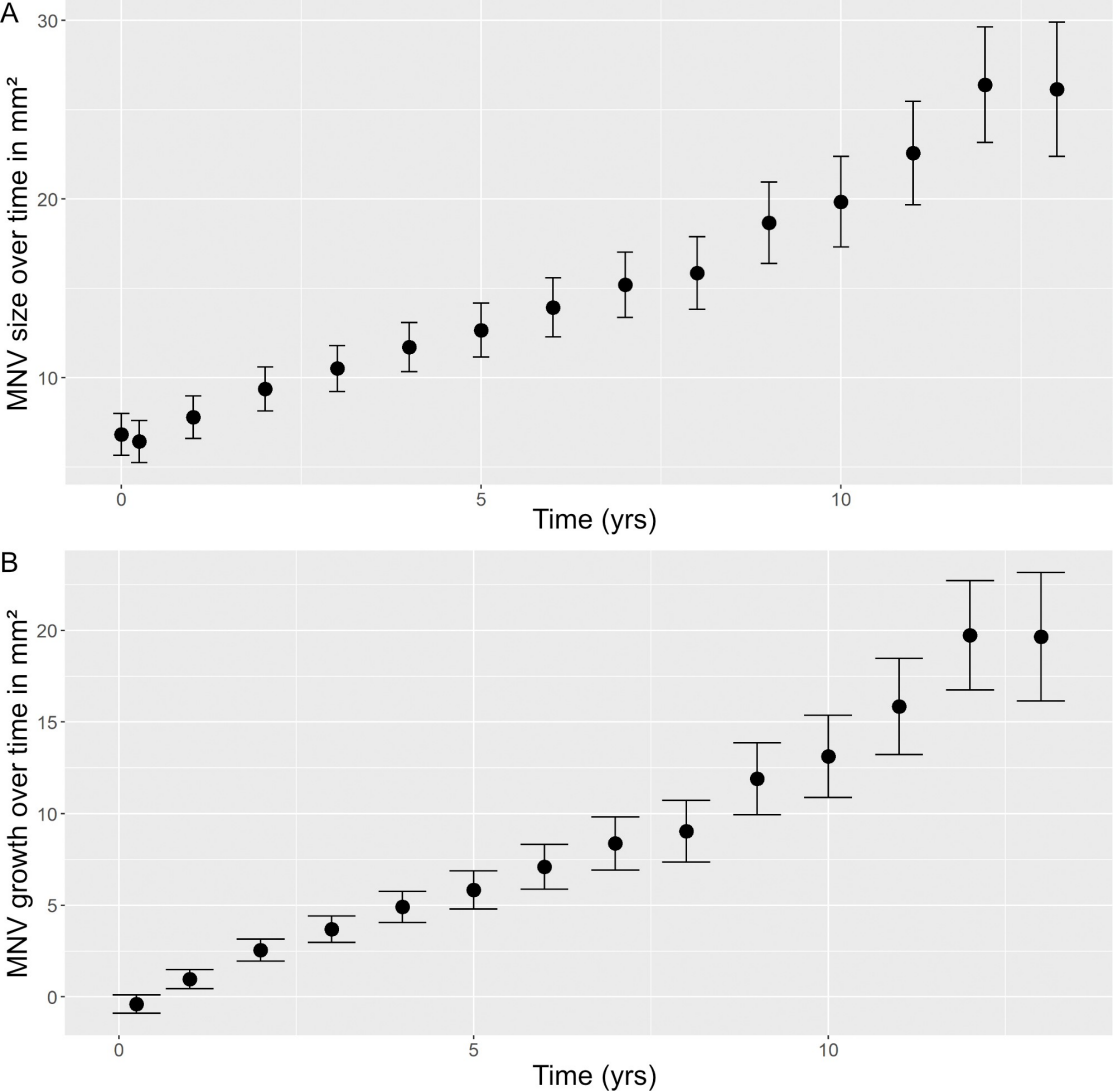
Change in macular neovascularization (MNV) size over time. Mean MNV size for each year of follow-up (A) and growth over time (B) are shown separately. Measurements for each point in time are presented as mean (spot) and error bars showing standard deviation. A clear trend of increase in MNV size over time can be observed, both in terms of mean MNV size and MNV growth. MNV = macular neovascularization.

Mean MNV size demonstrated a continuous increase over time (Fig 1A). To exclude the influence of different initial MNV size (the initial size ranged between 0.27–30.0 mm^2^, mean 6.7± 5.6 mm^2^, median 5.2 mm^2^) and thus quantify growth during follow-up, the difference between the MNV size at a specific year of follow-up and MNV size at year 0 (initial MNV size) was analyzed. This demonstrated continuous MNV growth during follow-up (Fig 1B). Based on those values, a yearly mean annual growth rate of 1.24 ± 1.65 mm^2^ / year was calculated (annual MNV growth at Table 2).

**Table 2:** Maculareovascularizationn growth over time. An increase in mean macular neovascularization (MNV) size per year of follow-up and MNV growth (difference between the MNV size at follow-up and initial MNV size) over time are shown. Based on these, the resulting growth per year was calculated.

	n eyes	mean MNV size (mm^2^)	initial mean MNV size of remaining patients at annual f/u (mm^2^)	MNV growth (size at respective visit–- size at baseline) (mm^2^)	mean growth rate per year (mm^2^)
initial	143	6.7	6.7	.	.
3 months	143	6.3	6.7	-0,4	-1.6
1 year	143	7.6	6.7	1.0	0.9
2 years	128	9.3	6.7	2.5	1.2
3 years	115	10.6	6.9	3,7	1.2
4 years	97	12.2	7.3	5.0	1.2
5 years	66	12.3	7.7	4.6	0.9
6 years	55	13.5	8.2	5.3	0.9
7 years	38	15.0	9.3	5.7	0.8
8 years	27	15.8	8.9	7.0	0.9
9 years	17	19.6	9.7	9.9	1.0
10 years	12	21.9	11.4	10.5	1.1
11 years	7	25.7	15.5	10.3	0.9
12 years	5	27.6	13.4	14.2	1.2
13 years	3	30.2	17.7	12.6	1.0

f/u = follow-up; MNV = macular neovascularization.

**Fig 2:**
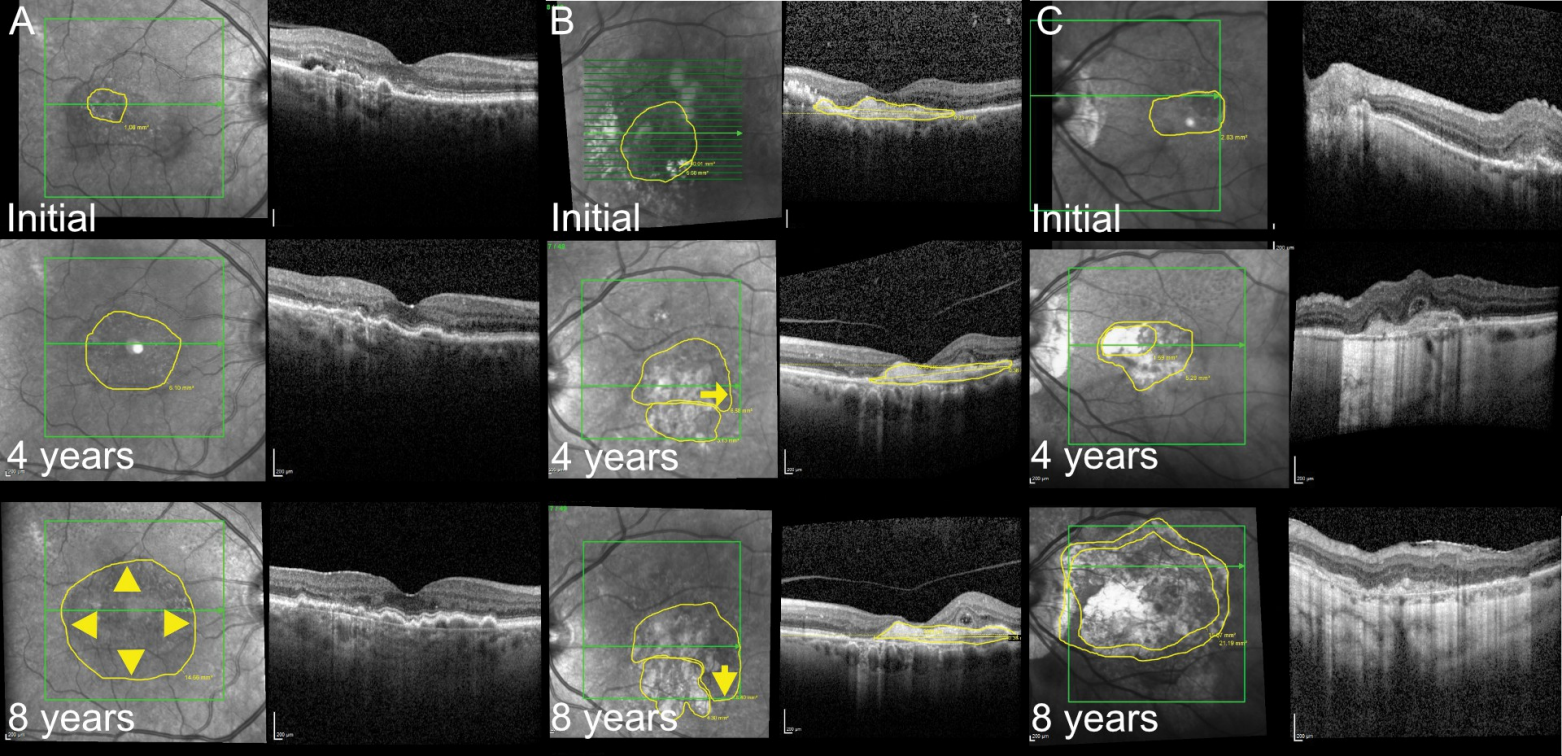
Macular neovascularization (MNV) growth patterns and atrophy development. A. “Centrifugal” MNV growth; Right eye of a 65 y/o female with 63 injections over 8 years. MNV size increases from 0,92 mm^2^ initially (top image), to 6.10 mm^2^ at year 4 (middle image) and 14.66 mm^2^ at year 8 (bottom image). An even growth across the whole diameter of the lesion is observed (arrowheads in bottom image). B. “Excentric”MNV growth; Left eye of a 79 y/o male with 61 injections over 8 years. MNV size increased from 5.68 mm^2^ initially (top image), to 9.71 mm^2^ (including 3.31 mm^2^ cRORA below the MNV, as shown by the lower yellow circle) at year 4 (middle image) and 12.70 mm^2^ (including 4.3 mm^2^ cRORA) at year 8 (bottom image). The lesion appears to be growing primarily towards its temporal margin (arrow in middle image) and then downwards in later stages (arrow in bottom image). C. „Centrifugal”growth with large cRORA; Left eye of a 78 y/o female with 48 injections over 8 years of follow-up. MNV size increases from 2.83 mm^2^ initially (top image), to 5.28 mm^2^ (including 1.59 mm^2^ cRORA, shown by the inner circle) at year 4 (middle image) and 21.19 mm^2^ (including 15.07 mm^2^ cRORA, again shown by the inner circle) at year 8 (bottom image). An even MNV growth is observed while a large area of cRORA develops in the center, eventually covering most of the MNV area. cRORA = complete retinal pigment epithelium and outer retina atrophy; MNV = macular neovascularization.

Distinct variations in the direction of growth were observed, with some MNV growing centrifugally like an enlarging circle (Fig 2A), while others showed eccentric growth in different directions (Fig 2B). A linear mixed model regression model showed a tendency towards lower MNV growth in men (p = 0.0618) compared to women and in type 2 MNV compared to type 1 MNV (p = 0.0705). However, no statistically significant correlation between age, sex, MNV subtypes or number of injections per year and MNV growth was observed (summary of variable analysis in Table 3). Comparing the difference between initial and last VA with MNV growth showed a significant correlation (BCVA (logMar) vs MNV size (mm^2^) -0.016; CI lower -0.027, CI upper -0.005; p = 0.0044)

**Table 3:** Impact of possible influencing factors on macular neovascularization growth. The impact of age, optical coherence tomography (OCT) macular neovascularization (MNV) type (1 to 3), sex and number of intravitreal injections on the change in MNV size (mm^2^). For MNV-type, type 1 was set as the reference, for gender, female was set as the reference. None of them show a statistically significant association with MNV growth.

Variable		estim. effect on change in MNV size	95%-CI		P-value	Initial Size
			lower	upper		
Age, yrs		0.0621	-0.07780	0.2021	0.3761	-0.06991 (-0.2443; 0.1044) p = 0.4237
MNV subtypes	type 2 vs type 1	-2.1995	-4.3177	-0.0813	0.0705	-0.1203 (-0.2976; 0.05697) p = 0.1785
	type 3 vs type 1	0.3104	-2.2938	2.9146	0.8114	
Sex	male vs female	-2.0042	-4.1113	0.1029	0.0618	-0.09273 (-0.2663; 0.08085) p = 0.2880
Injections per year		0.1664	-0.2277	0.5606	0.3998	-0.07373 (-0.2480; 0.1005) p = 0.3989

CI = Confidence interval; estim. = estimated.

### Development of cRORA

**Fig 3:**
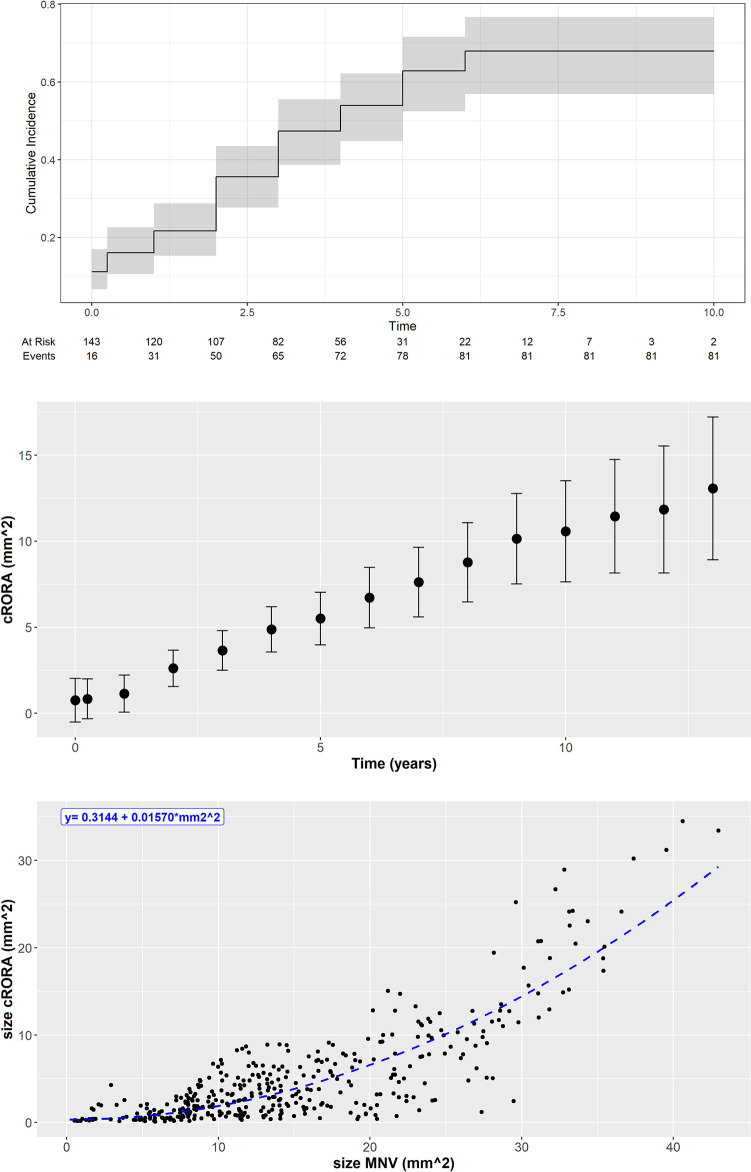
Development of complete retinal pigment epithelium and outer retina atrophy (cRORA) over time. A. Kaplan-Meier analysis showing the time-to-event of the development of cRORA. More than 60% of eyes had developed cRORA after 5 years of follow-up. B. Mean cRORA size over time. A clear increase in mean cRORA size can be observed over time. C. A positive relationship between individual MNV and mean cRORA size can be observed. This relationship is significant (p = 0.0001) and can be characterized by the formula cRORA size = 0.3144 + 0.01570 * (MNV size). cRORA = complete retinal pigment epithelium and outer retina atrophy; MNV = macular neovascularization.

Development of cRORA was frequently observed during long-term anti-VEGF treatment in the area overlapping with the MNV (see example in Fig 2C). Overall, 98 (68.5%) of the 143 eyes developed cRORA at some point during follow-up (Fig 3A). Analyzing the frequency of the presence of cRORA over time in eyes without initial cRORA (125 eyes) a continuous increase of relative presence was observed, with more than 50% of eyes showing cRORA after 4 year of follow-up and more than 60% showing cRORA after 5 years (Fig 3A). The longest follow-up of an eye without eventually developing cRORA was 10 years. Every eye with more than 10 years of follow-up thus developed cRORA (annual cRORA growth at Table 4).

**Table 4:** Development of complete retinal pigment epithelium and outer retina (cRORA) atrophy over time. Only the 125 eyes without any sign of cRORA at the commencement of anti-VEGF treatment were included in this subset. Over time, a significant increase in the number of eyes that exhibit cRORA can be observed and all eyes with follow-up of more than 10 years show cRORA by the end of the study. cRORA size increases over time, although yearly increases vary between 0.4 and 1.7 mm^2^ with a mean of 1.22 mm^2^ per year.

	n eyes	n (%) eyes without cRORA	n (%) eyes with cRORA	Mean cRORA size, mm^2^	Mean cRORA growth per year, mm^2^
initial	125	125 (100)	0 (0)	.	.
3 months	125	120 (96)	5 (4)	0,7	0,7
1 year	125	112 (90)	13 (10)	1,1	0,8
2 years	112	83 (74)	29 (26)	2,1	0,8
3 years	102	62 (61)	40 (39)	3,2	1,7
4 years	85	46 (54)	39 (46)	4,5	1,6
5 years	55	23 (42)	32 (58)	3,8	0,8
6 years	45	18 (40)	27 (60)	4,4	1,4
7 years	32	12 (37)	20 (63)	4,2	0,6
8 years	24	7 (29)	17 (71)	4,9	0,9
9 years	14	4 (29)	10 (71)	6,8	1,2
10 years	9	2 (22)	7 (78)	6,8	0,4
11 years	5	0 (0)	5 (100)	8,7	1,1
12 years	3	0 (0)	3 (100)	4,0	0,3
13 years	1	0 (0)	1 (100)	7,8	0,7

cRORA = complete retinal pigment epithelium and outer retina atrophy.

A continuous, linear growth of mean cRORA size over time was observed by a rate of 1.22 mm^2^ / year (Fig 3B). A statistically significant association of mean cRORA size with MNV size was observed (p = 0.0001), which was characterized by the equation: cRORA size = 0.3144 + 0.01570 * (MNV size)^2^ (Fig 3C). In order to calculate the growth rate of newly developing cRORA over time, only mean cRORA size at the annual visits (up to year 11) of the 127 eyes without any sign of cRORA at the begin of anti-VEGF treatment were analyzed (Table 4). This analysis demonstrated a continuous cRORA growth by a mean annual growth rate of 1.22 ± 1.3 (range 0.05–- 6.9, median 0.9) mm^2^ per year. Comparing the difference between initial VA and last VA with cRORA growth did not reveal a significant correlation (BCVA (logMar) vs MNV size (mm^2^) -0.012; CI lower -0.027, CI upper 0.004; p = 0.1343).

## Discussion

Our study demonstrates a continuous growth of mean MNV size under frequent anti-VEGF therapy (using IVAN treatment strategy guided by “activity” assessment based on fluid analysis on OCT volume scans). We also observed that cRORA developed in an increasing number of eyes over follow-up with cRORA present in 60% of eyes after 6 years and in all eyes that completed more than 10 years of follow-up. Once present, cRORA size also grew over time, at a similar rate as did MNV size (mean MNV growth = 1.24 mm^2^/year, mean cRORA growth = 1.22 mm^2^/year). This highlights that the underlying biological changes in nAMD (e.g. presumed hypoxia of the outer retina) [18] continue even under frequent anti-VEGF treatment.

Previous studies investigating type 1 MNV without exudation, i.e. “quiescent MNV”, often characterized by shallow, irregular RPE elevation (SIRE), also found that MNV grew over time, although these patients did not receive any treatment [9, 11]. The present study shows that MNV growth can also be observed in eyes under effective anti-VEGF therapy (at least from the aspect of anti-permeability and fluid control using the IVAN treatment strategy) [19]. Other studies using the “reactive” IVAN treatment strategies with 4 years of follow-up also showed MNV growth with a mean annual increase in MNV size of 0.8–0.9 mm^2^ [17]. While the same effect of MNV growth was observed in the present study, we saw a greater annual increase in MNV size. This may due to the limitations of the OCT-based MNV size delineation in our study. Future AI-based measurements of MNV size may help to get more “objective” MNV size measurements.

The study by Cozzi et al. [17] also assessed MNV growth in eyes that were treated using a proactive T&E treatment strategy. Those eyes showed no growth in MNV size during follow-up. This could suggest that the IVAN treatment strategy allows for intermittent spikes in VEGF levels and may thus be effective in achieving anti-permeability, but that these spikes are sufficient to induce continuous MNV growth. In addition, other factors independent of VEGF may also serve as angiogenic stimuli for MNV growth. For example, it was recently shown that factors such as fibroblast growth factor-inducible 14 (FN14) can stimulate MNV growth *in vivo* without significantly affecting VEGF levels [20]. Moreover, the higher percentage of patients developing type 1 MNV under anti-complement therapy in geographic atrophy trials may indicate that inflammatory factors are involved in angiogenesis, in addition to VEGF [21, 22].

Many long-term studies on anti-VEGF treatment in nAMD demonstrated a stabilization or minor deterioration of BCVA over many years, if permeability was suppressed effectively [2–4, 6]. These positive functional observations in combination with the present evidence of continuous MNV growth can be interpreted to show that, as is the case in non-exudative nAMD [9, 11], the presence of MNV alone has no general negative functional impact if fluid exudation can be “controlled”. This supports the concept that MNV may have a nourishing function for the overlying RPE and photoreceptors, as was previously proposed based on clinical [23] as well as histopathological [11] studies. Our data showing that greater MNV size correlated with a greater drop in visual acuity during follow-up and a higher likelihood of cRORA development also suggests, however, that once MNV are large and mature they may not be able to adequately nourish the RPE and photoreceptors anymore.

We also observed a large interindividual variation in MNV growth. This variation concerned both the speed of growth (slow and fast growing MNV) and the direction of growth (mostly centrifugal, sometimes irregular) and could not be explained by factors such as age, gender or MNV subtypes. This may indicate that the pathogenetic processes that cause and further maintain angiogenesis are greatly dependent on individual factors. In addition, the study protocol with annual measurements of MNV size mean that some measurements were performed between injections, immediately after a series, or before a new series of injections. Especially from OCTA studies it is known that MNV perfusion areas may differ significantly between different time points during anti-VEGF therapy [13]. The transformation and maturation of MNV and the regression of SHRM and development of fibrosis during follow-up may also contribute to varying individual MNV growth characteristics over time.

Furthermore, the mean increase in MNV size was independent of the number of anti-VEGF injections. This supports the strategy—as is known from functional studies—that early detection of exudative nAMD is beneficial to ensure the best outcomes [24] also regarding the final size of the MNV under therapy, because increased MNV size may result in poorer functional outcomes [25].

A major morphologic feature associated with MNV growth was the development of cRORA, which has been observed in many previous long-term evaluations of anti-VEGF therapy in nAMD [2–4, 6, 24–28]. Presence of macular atrophy increased over time in all studies, with a reported presence of 24.4% after 2 years in the IVAN trial [28], 59% of eyes after 5 years in the CATT-study [25, 26], between 37% [6] and 98% [2] (SEVEN-UP) after 6 to 7 years, and 89.5% after 7 years in the IVAN-study [4]. Sitnilska et al. could even find a continuous, yearly increase in the percentage of patients affected by atrophy over time, with 57.7% of patients showing atrophy at follow-up of slightly more than five years [29]. Some of the observed variation may be due to the methodology, since most studies were performed before the CAM classification came into effect and definitions of atrophy may therefore have differed. Based on the definition of cRORA, in eyes without cRORA at baseline we found we found a very similar incidence with 26% of eyes to show cRORA after 2 years, a 5-year presence in 58% of eyes, and a 7-year presence in 63% of eyes. Since our study also reported on an even longer follow-up of up to 14 years, we could also reveal that cRORA was present in all remaining patients after anti-VEGF treatment for more than 10 years. Since only very few patients completed this very long-term follow-up, larger studies are needed to validate this finding.

As is the case for MNV growth, cRORA growth also varies considerably within our cohort with a mean annual growth rate of 1.22 mm^2^. A study by Gune et al. using a similar OCT-based measurement method for macular atrophy reports a 2-year growth rate of 0.95 mm^2^ per year [30], while other studies reported lower annual cRORA growth rate of 0.4 mm^2^ during anti-VEGF therapy, which was independent on the treatment regimen (PRN or T&E) [31]. This lower growth rate may be influenced by smaller areas of macular atrophy on autofluorescence images compared to cRORA analysis on OCT images. The growth rate in geographic atrophy, by contrast, appears to be larger with an annual growth of 1.78 mm^2^ /year based on a review by Fleckenstein et al. of studies using color photograph or FAF analysis [32]. This difference was echoed by another study comparing macular atrophy in nAMD and geographic atrophy growth, which also found slower atrophy growth in MNV patients [33]. The reason for this difference in growth rates remains to be established.

The predominant factor that influenced cRORA size in our study was increasing MNV size. Reasons for the presence and increasing size of cRORA in nAMD were not evaluated in this study but may be associated with initial pigment epithelial detachment [34], maturation or fibrosis of central MNV parts with resulting photoreceptor and RPE death [35], fluid fluctuation or simultaneous ongoing geographic atrophy. Whether anti-VEGF therapy in itself may also causes cRORA development or this merely represents the natural cause of the disease, remains a matter of debate [28].

Our study has several limitations that may influence the described findings. The MNV measurements were based on OCT images rather than ICG or OCT angiography, which may impact MNV measurements. In addition, this study is a retrospective long-term analysis from patients who started anti-VEGF therapy as early as 2009. During this time, the criteria for retreatment switched from functional (BCVA) to more and more strict morphological (OCT fluid analysis) criteria, which may have influenced treatment intensity over time, and only the IVAN treatment regimen was applied with potential “spikes” in VEGF concentration. Additionally, the type of anti-VEGF agents varied between patients and also over time in individual patients and was not taken into account in our study, which could also impact MNV growth. Since patients did not have annual angiographic examinations or color fundus photographs, differentiation of MNV parts with more fibrotic transformation or presumed capillary growth was not possible [36]. Strengths of our study include the very long follow-up duration, a highly standardized grading performed by one, experienced retina specialist, the study of real-word patients rather than those from a controlled trial, and cRORA grading on OCT rather than on fundus photograph as performed in some earlier reports.

In conclusion, our investigation of MNV changes under long-term anti-VEGF therapy demonstrates ongoing MNV growth over several years using the IVAN treatment strategy. This supports the concept that MNV may be part of a physiological biological repair mechanism and could support RPE and photoreceptor function, if permeability can be controlled effectively by anti-VEGF therapy. Whether remaining low anti-VEGF levels or other factors independent of VEGF are responsible for MNV growth under therapy must be investigated in future studies. Simultaneously, a continuous increase in incidence and size of cRORA was observed over time. The individual functional implications and cause of this process also have to be defined in future studies.

## Supporting information

S1 Data (XLSX)
